# Indicators for the Data Usage Index (DUI): an incentive for publishing primary biodiversity data through global information infrastructure

**DOI:** 10.1186/1471-2105-12-S15-S3

**Published:** 2011-12-15

**Authors:** Peter Ingwersen, Vishwas Chavan

**Affiliations:** 1 Royal School of Library and Information Sciences, Birketinget 6, DK 2300, Copenhagen, Denmark; 2 Oslo University College, Pb 4 St. Olavs plass, 0130 Oslo, Norway; 3 Global Biodiversity Information Facility Secretariat, Universitetsparken 15, DK 2100, Copenhagen, Denmark

## Abstract

**Background:**

A professional recognition mechanism is required to encourage expedited publishing of an adequate volume of 'fit-for-use' biodiversity data. As a component of such a recognition mechanism, we propose the development of the Data Usage Index (DUI) to demonstrate to data publishers that their efforts of creating biodiversity datasets have impact by being accessed and used by a wide spectrum of user communities.

**Discussion:**

We propose and give examples of a range of 14 absolute and normalized biodiversity dataset usage indicators for the development of a DUI based on search events and dataset download instances. The DUI is proposed to include relative as well as species profile weighted comparative indicators.

**Conclusions:**

We believe that in addition to the recognition to the data publisher and all players involved in the data life cycle, a DUI will also provide much needed yet novel insight into how users use primary biodiversity data. A DUI consisting of a range of usage indicators obtained from the GBIF network and other relevant access points is within reach. The usage of biodiversity datasets leads to the development of a family of indicators in line with well known citation-based measurements of recognition.

## Background

Access to biodiversity data is essential for understanding the state of the art of biotic diversity and for taking informed decisions about sustainable use of biotic resources and their conservation. Among several impediments to publishing and discovery of primary biodiversity data is a lack of professional recognition [1]. An important incentive for scientists to publish research articles, monographs or conference papers is the explicit recognition that their work receives by means of citations from fellow scholars. Owing to the common but tacit conventions established in academic communities, scientists recognize the use of previous research, critically as well as in a positive sense, by adding references to such work into their publication text and list of references. References can be seen as a kind of normative payment [2]. The reference lists can easily be broken down into single units, whereby each reference turns into a citation that can be aggregated in many different ways, forming a wide range of citation impact indicators [3,4]. Typically, original academic articles, their references and the citations they receive are indexed in citation indexes, such as the Thomson-Reuters' Web of Science database [5]. These means of academic recognition and impact in science constitute the central indicators applied in the well established field of 'scientometrics'. Similarly, we believe that institutionalization of a Data Usage Index (DUI) [1] demonstrating impact of data publishing is feasible, even for a dynamic and complex network, such as the Global Biodiversity Information Facility (GBIF). So far, however, no metrics exist for data usage, especially biodiversity data usage, that recognize all players involved in the life cycle of those data from collection to publication. A set of DUI indicators is lacking [1]. We propose the indicators for the development of a DUI based on search events and dataset download instances - thus not based on traditional scholarly references and citations because no data citation mechanism now exists. As a spin-off, the DUI is also intended to provide novel insights into how scholars make use of primary biodiversity data in a variety of ways. Similar to scientometric analyses applying rank distributions, time series, impact indicators and similar calculations based on academic publications, the usage of primary biodiversity datasets leads to the development of a family of indicators and other significant metrics.

By applying instances of viewing, searching and downloading biodiversity dataset records, three characteristics are observable that differ from the use of publication references. First, in contrast to having fairly complete information on the nature of the original work (and its journal) citing a work, one has only limited knowledge of the internet protocol (IP) address that viewed, searched or downloaded dataset records, such as its location, that is, its geographic and institutional affiliation. We do not know who actually viewed or downloaded the dataset records. Second, we only know the data publisher's name and location. We do not know who in reality designed, collected and prepared the contents of the dataset and its records. The proposed DUI indicators are thus directly attributable to the academic institution rather than to the scholars behind it. Third, the basic unit in the proposed DUI is a biodiversity dataset record. Thus, we regard the dataset record as analogous to a journal article and the datasets as analogous to a journal. Biodiversity datasets are produced by data publishers. The latter may produce several datasets. As in similar scientometric analyses normalization is done by means of the basic analysis unit: here this is the dataset record.

## Why a Data Usage Index?

In line with the publication and citation behavior mentioned above, and as stated by Chavan and Ingwersen (p. 5 of [1]), "[the] DUI is intended to demonstrate to data publishers that their biodiversity efforts creating primary biodiversity datasets do have impact by being accessed, searched and viewed or downloaded by fellow scientists". All players and their host institutions involved in the data life cycle from collection of data up to its publication require incentives to continue their efforts and recognition of their contribution. In a scientific digital library and open access environment, such as that developed for bibliographic information in astronomy [6], usage is measured in a two-dimensional way. The straightforward way is to apply common scientometric indicators with respect to citation patterns and impact. However, this track is not yet feasible in the case of biodiversity datasets. There are no robust and universally accepted standards for data(set) citations in scientific papers and quantitative analyses of citations to biodiversity datasets will provide unreliable results (see also below). A second avenue is to define usage metrics, based on requests, viewing and downloading of research publications in the form of metadata, abstracts or full text via the astronomy digital library client logs [6]. The citation analysis avenue clearly refers to the authors' intellectual property in the astronomy papers. The usage metrics avenue may also include players responsible for the technical infrastructure presenting such properties. The usage impact could be shared and the distribution of credit would be the responsibility of the host institution: a digital library or a data publisher.

Thus, the proposed DUI for primary biodiversity datasets is initially intended to apply this second avenue of action, based on usage indicators extracted from the usage logs of the GBIF data portal [7] and later on other access point log data. This avenue constitutes phase one of three, implementing a universal DUI, as outlined in [1]. The proposed DUI is thus expected to make the dataset usage visible, providing deserved recognition for their creators, managers and publishers, and to encourage the biodiversity data publishers and users to: increase the volume of high quality data mobilization and publishing; further use of primary biodiversity data in scientific, conservation, and sustainable resources use purposes; and improve formal citation behavior regarding datasets in research.

When developing DUI indicators one needs to take into account the fundamental characteristics of datasets and their usage patterns. As academic publications indexed in traditional citation databases, such as the Web of Science [5], PubMed [8] or SCOPUS [9], entire datasets rarely become deleted from the databases currently contributing to the GBIF data portal [7] and other similar archives and publishing infrastructures. Their original records are rarely edited or erased; datasets can, however, be updated and grow in number of records over time or be modified or restructured. This characteristic of datasets is associated with the potential for change also observed in many web-based documents.

By applying the usage logs of the GBIF data portal [7], the DUI indicators are confined to that context. The usage as measured by searches or downloads of dataset records is detectable only within the coverage of the host system logs, as in a library. However, in contrast to a closed library log system a substantial amount of log data are publicly accessible from the GBIF data portal usage logs and are consequently open to indicator calculations. The properties of the GBIF-mobilized data usage indicators bridges between known scientometric indicators on impact and existing socio-cognitive relevance or social utility measures used in information retrieval studies [10], such as download events, recommendation and rating metrics. The dynamic nature of the GBIF network suggests that short analysis windows be used for the indicator calculations, such as semi-annually, monthly or less, and that the underlying data structures become frozen in logs for later reproduction of analyses.

## Citation versus usage behavior and analysis

The usage pattern of scientific publications is very heterogeneous and no definitive theory of reasons for giving a bibliographic reference (a citation theory) has been put forward. Although there are many different reasons for providing references in the scientific communication cycle [11], including negative, self-citations and namedropping [12], their potential bias in the various indicators is commonly neutralized statistically at higher aggregation levels [13]. We assume that this will also be the case for biodiversity data usage. Hence, we expect that similar statistical conditions are attributed to reasons for dataset interest or usage as to traditional citing behavior at higher aggregation levels.

Recently, novel citation indexing systems have been launched to compete with Web of Science (Thomson-Reuters) [5], for instance, the SCOPUS [9] service based on Elsevier and other publishers' publication data and Google Scholar [14]; the latter is primarily dedicated the citation networks of the web, the open access domain. None of these citation-based systems take into account in consistent ways scientific datasets as targeted objects for use in academic work. The main reason is the lack of a persistent and deep data citation mechanism [1]. The situation is similar in a web context. From the beginning of the web almost two decades ago impact indicators similar to the ones used in scientometrics were proposed for institutional websites and web pages, based on the link structures [15]. This sub-field to scientometrics is commonly named 'webometrics' [16]. Notwithstanding, to date the field does not deal with scientific datasets, probably again for the lack of an adequate data citation mechanism [1]. A very recent DataCite [17] initiative has been launched in late 2009 to attempt to generate a universal identifier of scientific datasets on the internet. The international consortium consists of 19 institutions and has so far minted over 1 million digital object identifiers (dois) for datasets.

In a few cases scientometric types of analysis have been applied in relation to biodiversity issues, for example on distribution of species over biodiversity profiles [18]. In relation to biodiversity datasets Piwowar and Chapman recently studied dataset sharing frequencies associated with journal impact factor and researcher impact [19].

Here, we first briefly outline the key characteristics of the GBIF Network, especially the data portal [7]. We then define and describe 14 selected absolute and normalized DUI indicators, which are exemplified and discussed in the following sections. Relative as well as weighted usage impact factors are then described and discussed, followed by future work and conclusions.

## The GBIF network: coverage and characteristics

Figure 2 in [1] depicts a simplified representation of the current GBIF network configuration of servers and their contextual datasets (see [1] for a detailed description and discussion of this infrastructure). The proposed first phase of the DUI indicator developments is based on data usage logs of the GBIF data portal. These provide general usage data on kinds of access and searches via IP addresses as well as download events of datasets accessible through the GBIF data portal, established in 2001 [7]. Currently (as of 5 September 2011), over 300 million records published by 344 data publishers, with the largest data resource containing 42.2 million records, are accessible through the GBIF data portal.

The GBIF data portal [7] is open access and selected elements of portal usage are already publicly available. Immediately, when entering the portal, one obtains access to three essential pathways to the up-to-date dataset contents:

A. Explore species: datasets sorted by kingdom/group of species and species;

B. Explore countries: datasets displaying record occurrence, organized alphabetically according to recorded national locations of species in datasets;

C. Explore datasets: datasets displaying number of records, organized alphabetically into dataset publisher, country of dataset origin by publisher (with record occurrence) and species recorded in specified country (sorted by kingdom).

Path A provides dataset information on species. Path B gives data on occurrence of different species located or recorded in individual countries. In all three paths, but in particular in path C, rank distributions are feasible that can be transferred into spreadsheets. As expected, the distributions of datasets and records show the ''long tail' phenomenon [20,21]: a few datasets in a country have many records, whereas many have few; this phenomenon also concerns publishers or species.

Aside from directly gaining access to the dataset volumes and distributions, the GBIF data portal also provides free access to search and downloading events for each publisher and associated datasets, which can be defined for specific time slots via the dataset entry, Path C, to the data portal. At present only a maximum of 250,000 searching events can be effectively analyzed online from the data usage logs. Semi-annual, monthly or less extensive analysis periods should therefore be applied. Only the current number of stored datasets and records potentially available for searching or downloading in the same time window may be elicited from the internal GBIF data portal log for immediate public online analysis. The extraction of the searchers' IP addresses, location and number of times they individually search the portal can only be performed by staff in charge of the GBIF data portal. In the examples below we concentrate on usage indicators that are feasible to calculate online by the public in open access mode. They are thus reproducible. In total, the GBIF data portal provides five dimensions of data, characterizing datasets that can be used in a variety of dataset usage analyses:

1. **The geographical dimension:** Publishers (all unique 344 publishers = world level)

a. Countries - names are controlled by GBIF

b. Regions - manual aggregation of countries is necessary

c. Academic institutions (author) publishing dataset. Institutional names are controlled

2. **Topical categories**

a. Species - taxa, searchable and names are controlled

b. Geo-location of species - country; 'map-area', that is, geographical area given by co-ordinates, holding names and number of species

c. Other relevant categorization - for example habitats (not consistent across datasets)

d. Types of datasets - for example with special features in set

3. **Time dimension**

a. Analysis periods for downloads and searches - weekly to semi-annual

b. Indexing/loading analyses (volume per time entity) - entry/update date

4. **Size of units** - commonly used for normalization purposes in indicators

a. Number of records in dataset (or other unit)

b. Number of datasets in geographical or topical unit

c. File size in bytes of unit

5. **Interest (a-b) and usage (c-d) event data**

a. Occurrence of searches - events and records searched

b. Occurrence of dataset details viewed - events and records viewed

c. Occurrence of downloads - events and records downloaded

d. Occurrence of taxonomy downloads - events and records downloaded

The events of viewing data publisher and dataset metadata belong to characteristics of a searcher's interests and are indeed available in the GBIF data usage logs. However, they cannot be applied in further calculations because they do not entail record viewing. Such events are thus regarded as bounces. Further, similar to analyses of scholarly citations, the usage analyses do not discriminate between different purposes of use of datasets and records, nor their actual usefulness to later research works in the cases of usage through downloads. The latter would require comparison between download volume by a user and his or her actual use in publications shown through direct references to the dataset(s) in question.

## The usage indicators

The preliminary set of indicators relies on counting various events of searching and downloading records from selected GBIF units in given time windows. By 'unit' we mean typical GBIF defined entities, such as individual datasets or data publishers at institutional or geographical level, or group(s) of species. Because the hierarchy of data record, dataset and data publishers is well established by GBIF as a return of a query to the system, as is the entity of species group or individual species, it is up to the analyst to define further suitable aggregation entities of such units. 'Searching' (and viewing) indicates interest, whereas 'downloading' signifies usage on the side of the visitor accessing the GBIF data.

The DUI indicators are constructed in the form of absolute metrics or indicators normalized according to stored volume of records, that is, providing impact measurements. In addition they may be calculated relative to something, such as to the average download volume of dataset records across all datasets (the world) or selected thematic datasets in the world or a country. This results in index values that are comparable within countries or themes. Finally, indicators can be normalized, related and weighted according to specific dataset profiles of institutions or countries. The latter (weighted) indicators lead to dataset Usage Crown Indicators, in line with similar impact indicators for scientific publications and citations [22]. They make comparisons across units - such as publishers, countries and themes - globally field normalized and fair. At present the number of records in a dataset is set to the current number of records, but it could be defined as the number of records at the end of the search or download observation window, or as the average of records detected over the window in question. Table 1 demonstrates the basic range of absolute DUI indicators and selected normalized ones.

**Table 1:** Basic Data Usage Index indicators for primary biodiversity data published through the GBIF network

	Formula	Indicator	Description
1	*s*(*u*)	Searched records	Number of records searched/viewed (by IP address) in unit
2	*d*(*u*)	Download frequency	Number of downloaded records from unit
3	*r*(*u*)	Record number	Number of records in (period; dataset(s); geographical and/or species unit)
4	*S*(*u*)	Search events	Number of different searches (by IP address) in unit
5	*D*(*u*)	Download events	Number of different downloads from unit
6	*N*(*u*)	Dataset number	Number of datasets in (period, geographical and/or species unit)
7	*s*(*u*)*/S*(*u*)	Search density	Average number of searched records per search event
8	*d*(*u*)*/D*(*u*)	Download density	Average download frequency per download event
9	*d*(*u*)*/r*(*u*)	Usage impact	Download frequency per stored record per unit
10	*s*(*u*)*/r*(*u*)	Interest impact	Searched records per stored record per unit
11	*d*(*u*)*/s*(*u*)	Usage ratio	Ratio of download frequency to searched records in unit
12	*D*(*u*)*/S*(*u*)	Usage balance	Ratio of download events to search events for unit (in %)
13	*U*(*u*)*/r*(*u*)	Usage score	Ratio of unique downloaded records (*U*) to record number (in %)
14	*I*(*u*)*/r*(*u*)	Interest score	Ratio of unique searched records (*I*) to record number (in %)

Indicators 1-3 (Table 1) are absolute measures and calculated at the lowest aggregation level, that is, at record level for a given unit. They are absolute indicators and inform about interest (indicator 1, referred to as *s*(*u*), where *u* indicates the unit and usage (indicator 2, *d*(*u*)) and record quantity (indicator 3, *r*(*u*)). They can be retrieved directly through publicly accessible online analysis of the GBIF logs and indicators 2 and 3 form the cornerstones of the DUI indicators concerned with usage impact and density (indicators 7-11). Indicators 1 and 2 can, in addition, be used for rank distributions of user institutions extracted from the IP addresses and the viewed/searched or downloaded records, that is, importing knowledge from a given dataset. This is similar to analyzing the citing publications to a specific (set of) publication(s) in scientometric analyses [23]. The alternative case of knowledge export to a given IP address across many datasets is also feasible - in both cases, however, this is available only to internal GBIF staff. Such analyses lead to behavioral investigations of the usage of biodiversity datasets in research. The record number (indicator 3) is similar to simplistic publication counting (productivity assessment) in scientometric analyses and often used as the primary normalization element (in indicators 9, 10, 13 and 14).

Indicators 4 (*S*(*u*)), 5 (*D*(*u*)) and 6 (*N*(*u*)) are also absolute measures calculated at a higher aggregation level, for example, at event or dataset levels, providing an indication of popularity. Such indicators may themselves appear as denominators in other normalized indicators, for example, in indicators 7 and 8. The dataset number (indicator 6) is useful in distributions across dataset publishers as well as over species or groups of species for observing the most researched or analyzed ones. Indicators 7-10 are the most important normalized indicators and signify average interest and usage density for a unit and, in particular, usage and interest impact (indicators 9 (*d*(*u*)*/r*(*u*)) and 10 (*s*(*u*)*/r*(*u*))). Search and download density indicators 7 (*s*(*u*)*/S*(*u*)) and 8 (*d*(*u*)*/D*(*u*)) inform about the averaged number of records searched by search event or downloaded by download event. It is thus possible to have a high download density (indicator 8) for a unit, say a dataset, but at the same time a low usage impact (indicator 9): over the span of an analysis window there may be few but very dense download events; however, when normalized for potential number of records downloadable from the dataset the actual number of records downloaded is small and the usage impact low.

The difference between indicators 9 and 10 is that the latter informs about the average number of records that are viewed/searched during a given time span. Indicator 9, on usage impact, is thus narrower or more pointed because it informs about the searched/viewed records that actually have been downloaded. Hence the usage ratio, indicator 11 (*d*(*u*)*/s*(*u*)), which calculates that proportion of searched and viewed records in a unit that have been downloaded during a given time period. This is an indicator of the volume of searched records that is found to be relevant to further scrutiny, and it can be associated with the usage balance (indicator 12, *D*(*u*)*/S*(*u*)) for the same unit or compared with other units' usage ratios or balances. Indicator 12 informs about how many search events that actually led to usage in terms of download events. There can be great differences between the usage ratio and balance depending on the quantity that becomes downloaded at each event for further use. Finally, the usage (and interest) scores (indicators 13 and 14) serve as important quality indicators. They are in line with the 'citedness' indicator used in citation analysis [3]. The usage score (indicator 13, *U*(*u*)*/r*(*u*)) informs about the proportion of unique stored records in a unit that has been downloaded at least once during a specific time period. Similarly, the interest score (indicator 14, *I*(*u*)*/r*(*u*)) informs about the unique records that have been searched and viewed at least once during an observation window. The higher the score the larger the proportion of published dataset records that is found useful at least once by fellow scholars. From citation analyses it is evident that the degree of citedness has substantial influence on the citation impact [3,24]. Indicators 13 and 14 can be captured from the GBIF usage event logs only by GBIF staff.

The number of datasets produced by a publisher at a given point in time (indicator 6) categorizes publishers into small (less than 10 entities), medium (10-100), large (100-300) and ultra-large (over 300). Searching can be divided into unique searches, that is, the first-time searches as defined by IP address, and loyal searches, that is, the searches repeatedly visiting a unit in a given observation window. The GBIF data portal [7] applies Google bounce statistics and its bounce percentage is approximately 66%. GBIF defines a loyal search as characterized by a specific IP address, which, after an interruption of activity of at least 30 minutes, reappears as a searching mechanism of the GBIF data portal. As is well known from other web-based search engine logs [25], one is confident only that the same IP address is active, not of who is behind the address. This is the reason for naming the visits as 'searches' and not as visitors or searchers. Loyal searches are of interest because they presumably lead to more intensive usage than sporadic and unique searches. This assumption can be tested empirically via the DUI indicators. The volume of datasets can be measured in number of records, as in Table 1; however, file size in megabytes is also useful as a measure, because records can vary hugely in size.

## Examples of DUI indicators

We illustrate the DUI indicators (Table 2) in the form of the indicators 1-12 (Table 1). Table 2 is based on a simplistic time series analysis of events in first and second half of 2009 (periods a and b). The calculations concern the small dataset publisher, Herbarium of University of Aarhus (HUA), which published two datasets (so indicator 6, *N*(*u*), is 2). The Ocean Biogeographic Information System (OBIS) and its 180 datasets represent a large dataset publisher. This publisher is analyzed over one month only, 1-31 December 2009. To illustrate indicators for a medium dataset publisher and an aggregation at country level, Table 2 also demonstrates characteristics for the larger Danish Biodiversity Information Facility (DanBIF), publishing 38 datasets, and for Denmark for the period 1 July to 31 December 2009 (period b). Owing to duplicates the Danish total record number is smaller than the total sum of the Danish publishers' records. The datasets actually used during the observation period are named *n*(*u*).

**Table 2:** Dataset indicator examples: record numbers as of 31 December 2009

Indicator	Formula	OBIS Dec09	DanBIF-09b	HUA-09a	HUA-09b	DK 2009b
Searched records	*s*(*u*)	2,092,927	5,682,095	2,299,133	7,328,160	13,010,255
Download frequency	*d*(*u*)	555,835	854,761	809,468	717,102	1,571,863
Record number	*r*(*u*)	11,140,298	4,995,544	259,077	259,077	4,836,771
Search events	*S*(*u*)	42,860	249,214	126,449	198,910	448,124
Download events	*D*(*u*)	601	4,486	2,059	1,710	6,246
Dataset number	*N*(*u*)	180	38	2	2	40
Datasets used	*n*(*u*)	171	36	2	2	36
Search density	*s*(*u*)*/S*(*u*)	48.83	22.80	18.18	36.84	29.03
Download density	*d*(*u*)*/D*(*u*)	924.85	190.54	393.14	407.44	251.66
Usage impact	*d*(*u*)*/r*(*u*)	**0.05**	**0.17**	**3.12**	**2.77**	**0.32**
Interest impact	*s*(*u*)*/r*(*u*)	**0.19**	**1.14**	**8.87**	**28.29**	**2.69**
Usage ratio	*d*(*u*)*/s*(*u*)	0.27	0.15	0.35	0.10	0.12
Usage balance	*D*(*u*)*/S*(*u*)	0.014	0.018	0.009	0.009	0.014

HUA, Herbarium of University of Aarhus; DanBIF, Danish Biodiversity Information Facility; DK, Denmark (DanBIF and HUA combined); OBIS, Ocean Biogeographic Information System. Bold indicates central impact and interest scores.

We immediately observe that regardless of length of analysis window the number of searched records and download frequency is substantial, supporting the need for a DUI. Download events are very low compared with the number of search events across all three publishers and periods; the usage balance between download and search events is consequently also low: only approximately 1-2% of the search events lead to direct downloading. Even though Table 2 does not mirror a comprehensive event analysis but merely serves as illustration, one can observe the extensive download density and the substantial search density across the publishers and time windows: 190-900 records are downloaded per download event and approximately 18-50 records are retrieved per search event.

The corresponding interest and usage impact factors inform about the average number of times each record stored by a publisher has been searched or actively downloaded. In both indicators a value greater than 1.0 implies that in principle all the dataset records on average have been searched or downloaded at least once during the analysis period. Note that the denominator *r*(*u*) is kept stable for each unit in the analyses above with the value of 31 December 2009. Thus, the displayed impact measures for the early 2009 HUA datasets (period a) are probably of conservative nature. In the simplistic time series illustration for HUA over the two semi-annual periods in 2009 (a and b), we observe a slight decrease in usage impact (3.12 to 2.77), but a substantial increase in interest impact (8.87 to 28.29) and a doubling of search density from 18 to 36 records per search event.

In case of OBIS, one of the GBIF thematic networks for marine biodiversity, Table 2 demonstrates a high ratio of dataset usage (171/180 = 95%) and high search and download densities although only analyzed during a 1-month time slot. The usage ratio is sizeable and signifies that 27% of the retrieved records were actually downloaded, although only 1% of the search events resulted in download events - the usage balance ratio. It is our opinion that these indicators possess high information value as to the properties of biodiversity dataset applications.

In the illustrative example in Table 2, the OBIS indicators cannot be compared directly with the indicators of the two Danish data publishers because the analysis windows are different. For instance, the OBIS usage impact factor at 0.05 is very low owing to the small analysis time slot (1 month) and the size of the denominator *r*(*u*), which presumably would increase at a lesser rate between updates of its datasets than, for instance, the number of downloaded records during the same time. Going back in time, for instance covering 6 months as done with the Danish data publishers, would probably increase all enumerators extensively, but decrease the record number only slightly. The usage impact factor might thus be estimated to be 0.30.

Another reason for precaution at direct comparative studies lies in the search and usage differences between dataset publisher profiles consisting of different collections of datasets and ranges of taxa/species categories or other thematic or topical features. Some biodiversity datasets holding specific themes or species might be more 'popular', that is search or usage-dense, than other datasets. This is similar to the phenomena of citation-dense versus citation-poor research fields in the academic publication world. Comparisons of units or themes that have less usage-dense datasets with those that have more usage-dense datasets may well be biased. This profile-dependency leads to the application of normalized, relative and weighted usage indicators described and discussed below in the section on relative and weighted indicators.

A third factor involved when comparing different institutions is associated with size. It is less meaningful to compare large data publishers (such as OBIS) with smaller publishers such as HUA or DanBIF, although this is done frequently in a variety of university ranking calculations. The fairest mode of comparison is to compare units approximately of the same sizes. In the calculations this can be done by applying a 'brute force' parameter, for example by multiplying the normalized, relative and weighted usage impact factor for the analyzed units by number of available records in millions [3,22]. This will automatically group the ultra-large data publishers aside from the other smaller units.

## Relative indicators

The indicators exemplified in Tables 1,2 are all absolute measures, including those that are normalized. Such indicators are most informative when measured relative to some relevant fixed point. For instance, the two HUA datasets together show a much higher usage impact than the total Danish usage impact for the same period (2009b; Table 2), which is diminished by the lower DanBIF impact across its 36 datasets. This produces an index value, in which 1.0 signifies the expected score of the comparative entity, that is, that absolute score, which each relative unit should attempt to reach or supersede, for example the Danish score. Commonly such fixed points consist of entities at high(er) aggregation levels, for example datasets compared with publisher; publisher with sets of publishers, for example in regions or countries; or countries or groups of species compared with the world scores of the various indicators. In principle all five indicator dimensions can be applied in different combinations to produce relative measures: geographical, topical or taxa, time, size and usage. We are hence in the position to characterize a given unit from various perspectives. This is in line with the recommended scientometric standards concerning research evaluation of institutions to make such assessments more robust, reliable and valid [26]. Given that each primary biodiversity dataset publisher commonly covers a range of species and taxa, such categories can be used as a comparative tool in the calculations across datasets, publishers and countries. To apply taxa and species distributions as elements in relative indicators requires that GBIF log data on species and taxa become elicited across publishers and datasets so that the indicators (Tables 1,2) can be applied to such units. The algorithmic and technical refinement required can be implemented.

However, given that the publicly available GBIF data portal logs currently allow public online data capture and analysis of the number of searched records or downloaded records and stored record number for each dataset produced by a publisher, one can calculate indicators for each dataset relative to those for the publisher over the same period of time. Similarly for the publishers in a geographical area, region or country: their absolute indicator scores are relative to that of the region or country - or indeed relative to the world average.

Table 3 illustrates two cases of relative indicators at different levels of aggregation: first, calculation of the relative usage impact factor (UIF) index for the two datasets produced by HUA covering the July to December period of 2009: the AAU Herbarium Database and the AAU Palm Transect Database; and second, acting as the second aggregation level in the calculations, the two datasets and HUA combined with the other Danish publisher, DanBIF, are analyzed for their UIF index scores relative to Denmark.

**Table 3:** Usage Impact Factors for two datasets and two publishers relative to Denmark's UIF

GBIF Units	**Record number** ***r*** **(*****u*****)**	**Download frequency** ***d*** **(*****u*****)**	Absolute UIF	Relative index UIF to HUA	Relative index UIF to DK
AAU Herbarium databases	110,357	716,772	6.50	2.35	20.31
AAU PalmTransect databases	148,720	250,330	1.68	0.61	5.25
HUA provider	259,077	717,102	2.77	1.00	8.66
DanBIF, provider	4,995,544	854,761	0.17	-	0.53
Denmark	4,836,771	1,571,863	0.32	-	1.00

Analysis period: July-December 2009 (GBIF Data Portal, 31 December 2009).

Although being slightly smaller in size of the two datasets, the AAU Herbarium dataset contributes most to the HUA dataset provider's absolute usage impact and its high UIF index score (8.66) relative to the Danish performance (Table 3; the two data publishers constitute the Danish GBIF dataset publishers). Because this analysis at present does not have information on other countries' UIF scores for that period it is not known whether the national result is good or bad at European or world levels. The foreseen comprehensive DUI may help in this respect.

The formula for UIF calculation relative to some other entity (world or region or country or 'map area' or species), and providing an index score, is:

Where the given unit is (*u*), *d* is the number of downloaded records, *r* is the number of stored records and *n* is the total number of units in the denominator. For Interest Impact Factor (IIF) calculation, *d* is replaced by the number of searched records *s*.

Other relative indicators are calculated following the same formula scheme. In the case that the unit in question concerns dataset(s) or publisher(s) dealing with specific (groups of) species the sums of the Download Frequency  and the Record Number  concerns all dataset records dealing with that specific (group of) species within a specified geographical area (such as world or region).

## Weighted relative usage indicators

Table 3 demonstrates how one dataset (AAU Herbarium Database) weights down the other HUA dataset in terms of usage impact, absolute and relative. In the DanBIF case the only one database is highly popular, whereas several datasets demonstrate very low usage impact. Such datasets contribute different weight to the aggregated indicators of their hosts. This principle of weighting is sound because it demonstrates that the 'dataset profile' for each publisher is individual and this fact should be taken into account when comparing publishers or other units consisting of several datasets. By not treating each dataset as equally strong (and thus not using simplistic average calculations) a far more fair comparison is established. (The weighted calculation follows the principle of ‘ratio of sums’, such as of sums downloads for all units over records or events. Larger datasets then weight higher than smaller ones. The opposite calculation principle (sum of ratios divided by the units) treats all units of the profile equally; larger dataset ratios then weight higher than smaller ones, regardless of dataset volume.) As stated above, some usage-dense or high-impact datasets (or species) will influence extensively the aggregated indicator outcomes of a unit (such as publisher or country). We observe the same phenomenon when universities are compared for citation impact. In direct comparisons universities specialized in citation dense disciplines and excluding disciplines from humanities, social sciences and some technology-dependent disciplines may well rank higher than more broad-based universities [3].

This weighting principle is carried over into comparative indicators, also named crown indicators [22]. If one wishes to compare HUA with DanBIF one may do that at country level, as demonstrated in Table 3. But in all fairness one could argue that because the Danish impact consists of very few datasets (40 at the time of calculation) and the profile of DanBIF is quite different from that of HUA one should rather compare the two publishers according to 'species dataset impact', not at national but at world level. This is exactly what the normalized, relative and species profile-weighted usage impact indicator attempts to do.

This indicator is based on the assumption that research institutions deal with datasets of species (or other recognizable bio-categories or themes) across their datasets. A given institution or publisher will thus have a species profile, defining their datasets. For each species for that institution (the unit), one may calculate the record number (*r*(*s*)) and the download frequency (*d*(*u*)), which will provide a usage impact factor (UIF) for each species/category in the profile and a weighted absolute UIF score of the institution or publisher according to its species profile. That weighted score is then compared to a similar 'global' one made of the same weighted profile across all institutions dealing with the same species. This global score is hence a 'shadow' of the unit under analysis and not biased by dominant profiles of dataset providers in large countries. The global score serves as baseline by providing the expected global UIF, which serves as denominator for calculating the final Usage Crown Indicator (UCI):

where *d*(*u*) is the total download frequency from unit (*u*); *p* the number of different species (*s*) in the unit profile; *U* is the weighted expected global UIF for that given species: 

where *n* = total number of units taken into account with respect to the species (*s*), for example constituting the world or a region.

The UCI may also work with taxa or other topical or thematic classification entities applied across the GBIF mobilized datasets. The UCI generates an index value. A value of 1.0 signifies that the unit's usage is on par with the global (regional) one - given the unit's individual species dataset profile.

## Conclusions and future work

We have argued for the establishment of a set of indicators dedicated the usage of primary biodiversity datasets. This area of scientometric research and studies is entirely new and regarded as a supplement to traditional publication and citation analysis in biological fields. So far, the DUI constitutes the only feasible indicators for the recognition of the publishers of the primary biodiversity datasets, because citation data of such datasets are difficult to elicit explicitly from scientific publications. The lack of persistent identifiers and standards for deep data citation for datasets and data records [1] prohibits traditional citation analysis of this kind of data.

By giving credit to the dataset publisher as institution, the involved players, that is, scholars who collected, prepared and intellectually organized the dataset and the people responsible for the presentation and technical infrastructure making available the dataset, may indirectly obtain recognition according to the data publisher's stipulations.

We have formalized and exemplified a range of 14 central normalized and non-normalized DUI measures and proposed relative and weighted usage indicators based on five dimensions of dataset features (geographical location, time, topic, size and usage). These core indicators can be calculated through the publicly available GBIF data portal log files. In addition, the absolute measures are based on data that can be applied to generate rank distributions of dataset records over datasets and publishers. This will be demonstrated in a later publication. Both the DUI and the distributions may contribute to further the understanding of dataset generation, usage and other behavioral traits.

Meanwhile, the internal GBIF data portal logs contain data that makes it possible to propose and develop sets of even more robust indicators, such as the UCIs that makes use of species profile-weighting as described above. We propose a GBIF data portal plan to establish a DUI of crown indicators associated with the 344 dataset publishers across defined species groups and thematic categories, covering selected periods of 2009-2010. The next step is to incorporate other biodiversity data access point logs so that DUI calculations are possible covering a larger portion of the entire dataset network.

At present there are certain limitations to the way the DUI can be used. Information on the users of datasets that are searched or downloaded is only available through calculations made on the log data by GBIF staff. A further development of the DUI framework is to make such information accessible to the public. Secondly, an annual impact report, such as the Journal Citation Report published by Thomson-Reuters, on basic dataset and publisher usage data would support the interest for further indicator development and robustness. The issue of reproducibility of usage analyses is central to the adoption, acceptance and usability of dataset usage indicators as part of the scientific life cycle. This implies that GBIF stores frozen snapshots of the portal data structure for future availability.

It is important to emphasize the utility of relative as well as weighted and robust usage indicators. As in the case of citation impact, the interest or usage scores of a given unit cannot stand alone but must be observed relative to a common and fair baseline, as we have proposed above concerning the UIF. Only in this way may one avoid the usual pitfalls experienced in citation and publication analyses carried out in simplistic and one-dimensional manner by unskilled researchers.

## Competing interests

The authors declare that they have no competing interests.
